# A sustainable artificial intelligence facilities management outsourcing relationships system: Case studies

**DOI:** 10.3389/fpsyg.2022.920625

**Published:** 2022-08-04

**Authors:** Ka Leung Lok, Albert So, Alex Opoku, Charles Chen

**Affiliations:** ^1^National Research Base of Intelligent Manufacturing Service, Chongqing Technology and Business University, Chongqing, China; ^2^Asian Institute of Built Environment, Hong Kong, Hong Kong SAR, China; ^3^College of Engineering, University of Sharjah, Sharjah, United Arab Emirates; ^4^College of Business and Information Technology, University of Phoenix, Hawaii, HI, United States

**Keywords:** artificial neural networks (ANNs), facilities management outsourcing relationships system, facilities management strategies, outsourcing categories, CORE model, sustainability

## Abstract

The purpose of this article was to validate the published artificial intelligence (AI) facilities management (FM) outsourcing relationships system by real business cases in the working environment. The research aims to inspire the modern FM professionals in different industries with some challenging and innovative concepts about FM outsourcing relationships between facilities owners and service providers. First, it will briefly introduce the theory of the FM outsourcing relationships system on how it can help the FM seniors and strategists to design their FM daily strategies wisely and make their business more effective and productive. Second, it will also introduce what the research is practically doing in the stage of case study for test and verification. It is concluded that FM outsourcing categorization may help to define the appropriate relationships. This further detailed outcome generated from the AI can be considered a solid reference to define and explain the existing outsourcing relationships between the stakeholders and the service providers to assign an outsourcing category to the FM relationship between the client and service provider based on the learnt rules.

## Introduction

Nowadays, no modernized people can escape the modern digitalization trend. All ages of life are necessary to learn and update the modern IT techniques with their IT applications. To have effective communication and productivity, people need to depend more on IT applications in daily life than before. No matter what kind of profession, the IT application can further improve the efficiency and productivity of the profession without a doubt. Lee et al. (2021) argued that digitalization has had a great influence on the way existing everyday functions are performed in various traditional industries and also new industry such as facilities management (FM). The decision support system for predictive maintenance of building facilities is utilized for artificial intelligence (AI), machine learning (ML), and predictive maintenance management systems (Villa et al., 2021). This new FM outsourcing relationships system can be beneficial to the FM community. However, the success of this outsourcing relationships system should not only depend on the efforts of the FM researchers but also most importantly on the application of the new system by the FM practitioners and community (Lok et al., 2020).

Sustainable FM can be related to building performance, sustainability tools and standards, user perception, satisfaction, productivity, and sustainability management (Nielsen et al., 2016). A study by Collins et al. (2019) that explored the gap between sustainable buildings and FM shows that there is a need to bridge the traditional gap between design, construction, and FM that demands more effective solutions based on life-cycle assessments. Insufficient data and information can affect the digitalized development of FM (Mannino et al., 2021). Although FM relationships can contribute to the success of FM outsourcing services, there is still neglect of the importance of FM relationships between clients and service providers (Lok and Baldry, 2015). The FM contingency outsourcing relationship (CORE) model can be used to quantitatively measure the current outsourcing performance of an FM service provider and evaluate the future outsourcing plans, abilities, and capacities of the service provider for specific FM contracts that comply with identified outsourcing relationships (Lok et al., 2021). This article aims to validate the sustainable AI FM outsourcing relationships system through real case studies. After the real data consolidation and validation process, this outsourcing relationship system is robustly applied to the FM outsourcing community with different applications in the environment.

Lok and Baldry (2015) addressed that the CORE model originated from the four outsourcing relationship types (FORT) model; the CORE model is used in the globalized FM industry, while the FORT model is originally used in the global information technology industry. After analysis of the simulated case studies of the four different categories (i.e., in-house, technical expertise, commitment, and common goals) of the CORE model from the perspective of the various clients, this study further builds on the previous work on the outsourcing relationships between clients and globalized FM service providers.

It further explores the application of this model with the aid of artificial neural networks (ANNs) toward a sustainable future. According to the CORE model, there are four original outsourcing categories, i.e., *OC*1, *OC*2, *OC*3, and *OC*4 (Lok et al., 2021). However, nothing is perfectly operated in the real world. After validation of the system by the raw case studies, it is necessary to introduce three new groups that are the in-between categories as outsourcing categories, i.e., *OC*1/2, *OC*2/3, and *OC*3/4, to further holistically explain the mechanism of the system.

A quantitative methodology through a survey is used to analyze a total of eight outsourcing strategies for the seven outsourcing relationships. A set of designed rules of the CORE has been introduced and discussed regarding the approaches to investigate the four simulated outsourcing relationship systems (Lok et al., 2020). This study investigates how the seven outsourcing categories can be systematically and efficiently implemented into the FM outsourcing relationships through the methodology of scientific AI in the real world. It can be concluded that FM outsourcing categorization may help to define the appropriate relationships. This detailed practical outcome generated from the ANN validation can be considered a solid reference to define and explain the existing outsourcing relationships between the stakeholders and the service providers. After understanding the outsourcing categories between the client and service providers with the research on FM relationships based on the learnt rules before, it is clear that the facility owners and users can accurately design and formulate their FM outsourcing strategies to improve their productivity of FM services much more efficiently and systematically.

## Materials and methods

As for exploring the opportunities of Industry 4.0 technologies, bringing such techniques as big data analytics, modeling, and simulation, this study is a validation of the published theoretical knowledge on AI FM outsourcing relationships system creating an essential competitive edge that generates real value for FM businesses. A quantitative methodology through a questionnaire survey is used to analyze eight outsourcing strategies for the four outsourcing relationships in first instance. This FM outsourcing relationship system is composed of four kinds of outsourcing categories through real-life practical case studies. On the design of data collection, each case study can be considered in alignment with a suitable outsourcing relationships category.

The data collection period was conducted from early August 2021 to the end of December of 2021, i.e., 5-month period. Initially, it was planned to have four case studies for the four outsourcing relationship categories, respectively, in this survey. During the period of the COVID-19 pandemic, it was rather difficult to collect the primary raw data in Hong Kong SAR, China, because of various industries with intermittent closed and lockdowns. To effectively collect the data, this survey was used to apply the strategic approach specifically on the sector of the construction and built environment where the FM professionals were more technically understanding of the scope of research.

There are two approaches to find out the suitable case studies of the companies or institutes. On the one hand, companies or institutes were invited by individual research connections. But, on the other hand, invitational emails have also been sent to the local FM or real estate professional networks such as the professional institutions. The questionnaire participants are mainly the FM facilities owners or users in the built environment practitioners to join this survey. Each respondent’s company or organization was required to invite two or three respondents to complete the survey in the same working environment on the standardized questionnaire. This study aimed to select and focus the participants in the built environment that as a result can be more robust and reliable. Those participants consider and accept the significance and importance of FM in their companies’ business. The practical case studies come from industries such as building maintenance, property management, property development, leisure and culture, education and exhibition, including local firms and international company.

The research analysis of the raw data by ANN was also undertaken in parallel with the data collection stage. The reason was that it was necessary to identify the outsourcing relationship category corresponding to the specific case study. After analysis of each case, there was an observation that some outcomes cannot exactly fall in the four outsourcing categories, i.e., *OC*1, *OC*2, *OC*3, and *OC*4, because such outcomes very often fall close to the border between two neighboring categories Therefore, it is essential to establish additionally three categories, i.e., *OC*1/2, *OC*2/3, and *OC*3/4, for pursuing a comprehensive FM outsourcing relationship system. Here, *OC*1/2 means a category lying between *OC*1 and *OC*2, and so on. Finally, there were a total of seven outsourcing categories for the system. The details are given in the analysis of the article.

## Criteria for the data collection stage

There are 10 criteria for the companies or organizations to join this research as case studies. (1) The company can be regional or international. (2) The company can be a facility owner or facility user. (3) The company can provide 2–3 respondents to complete the questionnaire individually without external support for fair measurement of the status. (4) The priority of target participants is the senior- or middle-level professionals required from the regional or international clients or companies. (5) The proposing target participants do not need to have any idea about what category their company belongs to. (6) The proposing target participants have been briefed before commencing to complete their questionnaires. (7) The proposing target participants can cross-check with an independent standard belonging to his/her company. (8) Different people of the same company should compromise the outcome with each other by the fair, objective, and reliable method. (9) ANN can produce a satisfactory and scientific result. (10) ANN analysis does not allow any major parameter out of eight outsourcing strategies as equivalent to zero. If there are no inputs of figures in the ratings of the questionnaire, it is necessary to assign “1” to the outsourcing strategy and the weight to “0.” Then, it has no impact on the overall assessment.

## Analysis of the data

### The artificial neural network for outsourcing categorization and generation of final outsourcing category

The whole ANN structure was previously discussed in detail in Lok et al. (2021) regarding how to use the ANN with more quantitative input. After further thorough investigation, this article discusses an application of ANN to the CORE through a detailed mathematical mechanism by using the bespoke formulae and raw quantitative data of case studies. There are two axes for the quantitative measurement of the proposed FM outsourcing strategies, i.e., *X*-axis and *Y*-axis, of the eight outsourcing strategies for the four outsourcing relationships (Lok et al., 2020). Accordingly, the *X*-axis is used for measuring the influence of the outsourced FM portfolio on client’s competitive position and long-term plan, whereas the *Y*-axis is used for measuring the ownership and control of various FM assets transferred to the service providers. It can predict the outsourcing relationships of the FM stakeholders of the specific FM outsourcing contracts such as building maintenance, cleaning, security, and catering. There are four inputs (*OC1*, *OC2*, *OC3*, and *OC4*) to the ANN and one output (*OC*). *OC1-4* was computed from raw data surveyed in different FM firms, while the final value of *OC* was used to determine the outsourcing category.

Once the eight FM outsourcing strategies described have been filled in, eight values, all capped with the range [0%, 100%], including substitution of ownership (*SO1* and *SO2*) and substitution of control (*SC1* and *SC2*) on the *Y*-axis and competitive position (*CP1* and *CP2*) and long-term plan (*LP1* and *LP2*) on the *X*-axis, from the FM CORE model (Lok and Baldry, 2015, 2016). These eight values are further converted into four values of outsourcing categories, also within the [0%, 100%] range with reference to the mapping (Lok et al., 2020), according to the following four equations based on the Cobb–Douglas production function (Cobb and Douglas, 1928), where the indices of high = 3, medium = 2, and low = 1.


$$OC1=(SO1^{3}*SO2^{2}*SC1*SC2*CP1^{3}*CP2^{2}*LP1*LP2){}^{\frac{1}{14}}$$

$$OC2=(SO1^{2}\times SO2^{3}\times SC1^{2}\times SC2\times CP1^{2}\times CP2^{3}\times LP1^{2}\times LP2){}^{\frac{1}{16}}$$

$$OC3=(SO1\times SO2^{2}\times SC1^{3}\times SC2^{3}\times CP1^{3}\times CP2^{2}\times LP1^{3}\times LP2){}^{\frac{1}{18}}$$

$$OC4=(SO1^{2}\times SO2^{2}\times SC1^{3}\times SC2^{3}\times CP1^{2}\times CP2^{2}\times LP1^{3}\times LP2^{3}){}^{\frac{1}{20}}$$

These four values of outsourcing categories are fed into the well-trained ANN as discussed in Lok et al. (2021). As each input is from 0% + to 100%, it is converted to a range of [0.5, 5.5] by dividing the raw value of each outsourcing category by 20 and then adding 0.5 to the result. It is because when the ANN was trained, each outsourcing category was assessed in five grades, from 1 to 5. Here, for example, the raw range [0%, 20%] is mapped to a processed range [0.5, 1.5]. Then, the output is confined within the range [0.5, 4.5] as given by the expert database during the learning process because there are four outsourcing categories, i.e., *OC1*, *OC2*, *OC3*, and *OC4*. The final output category is determined by the following formula:


O⁢C={1  if⁢    0.5≤⁢O⁢C< 1.52 if⁢     1.5≤⁢O⁢C< 2.53 if⁢      2.5≤⁢O⁢C< 3.54 if⁢      3.5≤⁢O⁢C< 4.5

This part is to simulate the four outsourcing examples respectively with clear explanation of the framework. Lok et al. (2020) mentioned that there are four categories, i.e., *OC1, OC2, OC3*, and *OC4*, but in the real world, it is unreasonable to assign *OC1* to a company with a score 1.49 and *OC2* to another company with a score 1.51. Therefore, it is necessary to create three new categories, i.e., *OC1/2, OC2/3*, and *OC3/4*.

The real world is continuous, not discrete. The margin suggested to use is 0.15, i.e., transient zone = ±0.15. This study does not only include pure *OC1, OC2, OC3*, and *OC4* cases; there are a couple of marginal cases, e.g., *OC1/2* marginally between *OC1* and *OC2*, *OC2/3* marginally between *OC2* and *OC3*, and *OC3/4* marginally between *OC3* and *OC4*. By this modification, the ranges of the seven outsourcing categories now become *OC1* = [0.5000, 1.3499], *OC1/2* = [1.3500, 1.6500], *OC2* = [1.6501, 2.3499], *OC2/3* = [2.3500, 2.6500], *OC3* = [2.6501, 3.3499], *OC3/4* = [3.3500, 3.6500], and *OC4* = [3.6501, 4.5000].

## Discussion

Lok et al. (2020) explained that the relationship between the client and service provider belongs to the outsourcing category 1 (*OC*1) as a support/in-house group [high impact on (*SO*1) hard FM on the challenge of flexible facilities and (*CP*1) competitive advantage]; outsourcing category 2 (*OC*2) as the alignment/technical expertise group [high impact on (*SO*2) soft FM on the challenge of flexible relationships in service provision and (*CP*2) value points for leveraging FM portfolio and business process improvement]; outsourcing category 3 (*OC*3) as reliance/commitment group [high impact on (*SC*1 and *SC*2) managerial control and decision-making over operations, planning, development, and implementation of facilities and personnel replacement in-house FM personnel, (*CP*1) competitive advantage, and (*LP*1) competitiveness]; and outsourcing category 4 (*OC*4) as the alliance/partner group [high impact on (*SC*1 and *SC*2) and (*LP*1 and *LP*2) long-term competitiveness, a close partnership, strategic inter-organizational relationship, and new revenue], respectively. Relevant results of cases studies are recorded in the Supplementary Tables 1, 2.

### Analysis of outsourcing category 1

The *OC*1 is from case study 5, the public sector (education and exhibition) operating regionally with the number of employees <50. Its business nature is the provision of teaching and learning platform about the sustainable environment to the local community. The FM outsourcing services provider is in charge of the number of subcontractors providing the FM outsourcing services, including cleaning, building maintenance, and catering services. The two respondents of the survey are on the manager level. The outputs (raw) from the two respondents are 1.054 and 1.147, respectively.

Another *OC*1 is from case study 8, the private sector (property management) regionally. The number of employees is from 100 to 200. Its business nature is the provision on property management services. The FM outsourcing services provider is in charge of a number of subcontractors providing the FM outsourcing services, including security, cleaning, and renovation. The two respondents of the survey are associate director and senior officer level. The outputs (raw) from the two respondents are 1.058 and 1.107, respectively.

It is noted that analysis of *OC*2 is missing. Case study for *OC*2 is absent as the research team could not find an appropriate company that exactly falls within this category. Although this is not desirable, all cases were randomly chosen in the study and there was no loss of generality.

### Analysis of outsourcing category 3

The *OC*3 is from case study 2, international private sector organization with property development business services. The number of employees ranges from 500 to 1,000. The FM outsourcing services provider is in charge of building and electrical and maintenance works, office supporting, hygiene, and security system providing the FM outsourcing services, including office general decoration/fixtures maintenance, IT hardware, cleaning, and security. The two respondents of the survey are on the senior officer level. The outputs (raw) from the two respondents are 3.261 and 3.487, respectively.

### Analysis of outsourcing category 4

The *OC*4 is from case study 3, public sector organization (leisure and culture of government) operating regionally with the number of employees <50. Its business nature is a provision of quality cultural services commensurating as a world-class city and events capital. The FM outsourcing services provider is in charge of building management such as building patrol, visitor inspects, emergency response, vacuum, sweep and mop floors, trash cans emptying, and antimicrobial coating spray providing the FM outsourcing services, including security, cleaning, and antimicrobial. The three respondents of the survey are on the manager level. The outputs (raw) from the three respondents are 3.889, 3.875, and 3.907, respectively.

New outsourcing categories are necessary to be established because of several possible marginal cases in-between the four existing outsourcing categories. In the real world, the four existing categories are not inclusive enough to explain and interpret all comprehensive real cases. It is suggested that three new outsourcing categories 1/2, 2/3, and 3/4 can further explain and supplement the original identified outsourcing categories, i.e., *OC*1, *OC*2, *OC*3, and *OC*4. In summary, the characteristics of the new three groups are introduced as follows.

### Analysis of outsourcing category 1/2

The *OC*1/2 is from case study 1, a private sector (building maintenance) operating regionally with the number of employees <50. Its business nature is the provision of maintenance services to residents. The FM outsourcing services provider is in charge of repair and maintenance on building assets providing the FM outsourcing services, including maintenance works and services. The two respondents of the survey are on the manager level. The outputs (raw) from the two respondents are 1.573 and 1.449, respectively.

### Analysis of outsourcing category 2/3

The *OC* 2/3 is from case study 4, a public sector organization (construction and maintenance) operating regionally. The number of employees is 500–1,000. Its business nature is the provision of construction and maintenance services to districts. The FM outsourcing services provider is in charge of general property management services providing the FM outsourcing services, including security and cleaning services. The two respondents of the survey are on the property officer level. The outputs (raw) from the two respondents are 2.529 and 2.478, respectively.

### Analysis of outsourcing category 3/4

There are two cases identified in this category. The *OC*3/4 is from first case study 6, the public sector (property management) operating nationally. The number of employees is 500–1,000. Its business nature is the provision of housing supply services. The FM outsourcing services provider is in charge of building maintenance works providing the FM outsourcing services, including security, cleaning services, and customer services. The two respondents of the survey are on the supervisor’s level. The outputs (raw) from the two respondents are 3.695 and 3.410, respectively.

The *OC*3/4 is from second case study 7, the private (building surveying consultancy) operating internationally. The number of employees is 50–100. Its business nature is provision on building surveying consultancy. The FM outsourcing services provider is in charge of building management providing the FM outsourcing services, including cleaning and maintenance services. The two respondents of the survey are on the building surveyor level. The outputs (raw) from the two respondents are 3.370 and 3.434, respectively.

## Conclusion

IFMEC (2018) suggests that the FM sector has done well by adopting relevant technologies such as AI and Internet of Things (IoT) as part of the sector’s smart building agenda to support the realization of UN’s Sustainable Development Goals (SDGs), SDG 9 (industry, innovation and infrastructure) in particular. The digitalization of FM in terms of outsourcing is rather new in parallel with the industrial revolution 4.0. The FM services in case studies presented in this article include more on real estate services and less on soft user services. Opoku and Lee (2022) emphasized that partnership is an important factor in one of the 17 goals. They suggest that the FM sector should be at the heart of the engagement and drive toward integrating sustainability into daily FM practice to bring improvement in customer service. The concept of sustainable FM also brings together two concepts of FM and sustainable development by adopting technology and innovative business practices that could balance the social, economic, and environmental impacts of business decisions.

The captioned FM outsourcing relationship system mentioned in this and previous papers can contribute to the three sustainable aspects. The application of our well-trained ANN on eight real cases verified this methodology while the actual performance of each case was in line with the final outsourcing category obtained from the ANN. The significance of the decision of the outsourcing category of the FM outsourcing contracts is to continuously improve and enhance the FM outsourcing services delivered by the FM service providers. The value of the CORE model is used to evaluate and manage the FM outsourcing relationships of the FM service providers on sustainable environmental, social, and economic performance effectively and efficiently for short- and long-term FM plans for improving outsourcing performance. The contribution of this article is the quantitative and objective input of parameters of the testing firms. This implies that the facility owners can refer to this CORE model to systematically and effectively measure and record their outsourced facility goods and services. It can generate a big database for further analysis and evaluation. The productivity of the facility owners can also be increased and cost of the FM contracts can be reduced by application of our mechanical and systematic mechanisms.

The research limitation of this study is that it is rather difficult to identify adequate number of companies to join the case study. There were only eight case studies comprehensively conducted. The limitation of using ANN as a research method is that as more and more cases are studied, the infrastructure within it may slightly change due to the intrinsic learning ability of ANNs. This may also be an advantage because we are now using AI. ANNs help to gather all expert advice provided in the form of rules and then assess input retrieved from companies to calculate the final OC value, which is mainly a summary of all rules involved. Facility owners are also required to spend extra efforts and resources to identify the existing outsourced facility goods and services through the input, and this may require more time to train up the ANN before any commercial implementation.

The credibility of the proposed study can be further increased if more samples from real-life cases can be obtained from practitioners and more structured interviews can be undertaken with FM experts. This study does not investigate the financial elements within FM outsourcing contracts as they are usually highly confidential and sensitive. In the future, the model can be used to more accurately evaluate and predict how the outsourcing category of the FM outsourcing services can be determined by quantitative on-site measurement, and AI-based calculation and analysis.

## Data availability statement

The original contributions presented in this study are included in the article/Supplementary material, further inquiries can be directed to the corresponding author.

## Author contributions

KL: project designer. AS: research method designer. AO: project editor. CC: resources provider. All authors contributed to the article and approved the submitted version.

## Conflict of interest

The authors declare that the research was conducted in the absence of any commercial or financial relationships that could be construed as a potential conflict of interest.

## Publisher’s note

All claims expressed in this article are solely those of the authors and do not necessarily represent those of their affiliated organizations, or those of the publisher, the editors and the reviewers. Any product that may be evaluated in this article, or claim that may be made by its manufacturer, is not guaranteed or endorsed by the publisher.
