# Supplementary material for: A sustainable artificial intelligence facilities management outsourcing relationships system: Case studies
Source: Front Psychol. 2022 Aug 4;13:920625. doi: 10.3389/fpsyg.2022.920625 (PMC9423375; doi:10.3389/fpsyg.2022.920625)
Supplement: Supplementary file 1 [file Data_Sheet_1.doc]

Table 1 – Profile of details of the eight case studies of the seven outsourcing categories (Data Collection period: 20/8/21 to 5/12/21; 3.5 months)

| **Case Study** | **Sector/Size/ Number of employees/ Number of respondents/**  **Level of respondent/**  **Business nature** | **FM Outsourcing services** | **Nature of FM services providers of the case** | **Final raw output** | **Confirmed (*OC*)** |
| --- | --- | --- | --- | --- | --- |
|  |  |  | **Characteristics of Outsourcing Category** |  |  |
| 1 | Private (Building maintenance)/ regional/ Below 50/2/ Assistant Manager/ Provision on Maintenance services to residents | Maintenance works; services | Repair and maintenance on building assets   - The Outsourcing Category 1/2 (*OC*1/2) is that the relationship between the Client (C) and Service Provider (SP) belongs to a group in-between support/inhouse and SP’s alignment/technical expertise. - To justify this new group, it is essential to analyse the SO1 and SO2 of the Ownership Substitution of FM Outsourcing strategies. The impacts of SO1 and SO2 as in-between high and medium. As for the strategic influence of FM Outsourcing strategies, the impacts of CP1 and CP2 as in-between high and medium. - When will the company fall on the Outsourcing Category 1/2 (*OC*1/2)? If the company is dynamically and marginally categorized at *OC*1, it is still having a progression to *OC*2. If this is the case, we would consider this company at the Outsourcing Category 1/2 (*OC*1/2) (in-house/ Technical expertise) which has a high impact on (SO1) hard FM—the challenge of flexible facilities and (SO2) soft FM—the challenge of flexible relationships in service provision, but also has a high impact on (CP1) competitive advantage and (CP2) value points for leveraging FM portfolio and business process improvement. - The SPs have the *OC*1 and *OC*2 characteristics as follows. The SPs can provide an in-between high standard of mutually agreed equipment for confirmation of daily operations to the Cs’ requirements and can maintain good relationships with the Cs on each day. E.g., diligent work, professional manner, warm hospitality, close co-ordination and understanding the needs of customers. - The SPs also can provide in-between medium standards of tailor-made professional knowledge for the building assets and can maintain a general level of customer’s general routine productivity on a general level for monthly operations, including security management, waste management, cleanliness of the workplace and indoor environmental quality. | 1.573  1.449 | 1/2 |
| 2 | Private (Property development)/ International/500 – 1000/2/ Senior officer/ Provision on property development and management services | Office Maintenance; IT Hardware; Cleaning and Security | Builder; E&M works; Office Supporting; Hygiene and Security system   - The Outsourcing Category 3 (*OC*3) is that the relationship between the C and SP belongs to a group in SP’s reliance/commitment. - The impacts of SC1 and SC2 of Ownership substitution and CP1 and LP1 of Strategic Influence of FM Outsourcing strategies as quite high. - The SPs can provide at least sufficient or even a higher standard of mutually agreed working timeframe for work orders and progress of concern for confirmation of monthly operations to the Cs’ requirement and can also maintain good relationships with the Cs each day. E.g., diligent work, professional manner, warm hospitality, close co-ordination and understanding the needs of customers. - SPs can maintain monthly performance and value of customers’ satisfaction concerning health, safety, maintenance and operation management. | 3.261  3.487 | 3 |
| 3 | Public (Leisure and culture of Government)/ regional/ Below 50/3/ Manager/ Provision on quality cultural services commensurating as a world-class city and events capital | Security; Cleaning and Antimicrobial | Building management: Patrol buildings, inspect visitors, response to emergency; Vacuum, sweep and mop floors; empty trash cans and Spray antimicrobial coating   - The Outsourcing Category 4 (*OC*4) is that the relationship between the C and SP belongs to a group in alliance/partner. - The impacts of SC1 and SC2 of Ownership substitution and LP1 and LP2 of Strategic Influence of FM Outsourcing strategies as high as that of Outsourcing Category 3 (*OC*3). - The SPs can maintain yearly performance and value of customers’ satisfaction on emergency planning, business continuity and sustainability. | 3.889  3.875  3.907 | 4 |
| 4 | Public (Construction and Maintenance)/ regional/500 – 1000/2/ Property Officer/ Provision on construction and maintenance services to districts | Security; Cleaning | General property management services   - The Outsourcing Category 2/3 (*OC*2/3) is that the relationship between the C and SP belongs to a group in-between SP’s alignment/technical expertise and reliance/commitment. - To justify this new group, it is essential to analyse the SO2, SC1 and SC2 of the Ownership Substitution of FM Outsourcing strategies. The impacts of SO2, SC1 and SC2 as in-between high and medium. As for the strategic influence of FM Outsourcing strategies, the impacts of CP1 and CP2 as in-between high and medium as Outsourcing category 1/2 (*OC*1/2). The impacts of LP1 as in-between high and medium. - When will the company fall on the Outsourcing Category 2/3 (*OC*2/3)? If the company is dynamically and marginally categorized at *OC*2, it is still having a progression to *OC*3. If this is the case, we would consider this company at the Outsourcing category 2/3 (*OC*2/3) (Technical expertise/Commitment) which has a high impact on (SO2) soft FM—the challenge of flexible relationships in service provision and (CP2) value points for leveraging FM portfolio and business process improvement, but also has a high impact on (SC1 and SC2) managerial control and decision making over operations, planning, development and implementation of facilities and personnel replacement in-house FM personnel, (CP1) competitive advantage and (LP1) competitiveness. - The SPs have the *OC*2 and *OC*3 characteristics as follows. The SPs can provide in-between high and medium standards of tailor-made professional knowledge for the building assets and can provide an in-between high and medium standard of mutually agreed working timeframe for work orders and progress of concern for confirmation of monthly operations to the Cs’ requirement. SPs can also maintain monthly performance and value of customers’ satisfaction concerning health, safety, maintenance and operation management. - The SPs can also provide at least sufficient or even a higher standard of mutually agreed working timeframe for work orders and progress of concern for confirmation of monthly operations to the Cs’ requirement and can also maintain good relationships with the Cs each day. E.g., diligent work, professional manner, warm hospitality, close co-ordination and understanding the needs of customers. SPs can also maintain monthly performance and value of customers’ satisfaction concerning health, safety, maintenance and operation management. | 2.5292.478 | 2/3 |
| 5 | Public (Education and exhibition)/ regional/ Below 50/2/ Manager/ Provision on teaching and learning platform about the sustainable environment to the local community | Cleaning; building maintenance and catering | FM Management contractor in charge of number of subcontractors   - The Outsourcing Category 1 (*OC*1) is that the relationship between the C and SP belongs to a group in support/inhouse. - The impacts of both SO1 of Ownership Substitution and CP1, Strategic Influence of FM Outsourcing strategies as quite high. - The SPs can provide at least sufficient or even a higher standard of mutually agreed equipment for confirmation of daily operations to the Cs’ requirements and can maintain good relationships with the Cs on each day. E.g., diligent work, professional manner, warm hospitality, close co-ordination and understanding the needs of customers | 1.0541.147 | 1 |
| 6 | Public (Property management)/ national/500 – 1000/2/ Works supervisors/ Provision of housing supply services | Security, cleaning, customer and maintenance services/ General Building maintenance works | Cleaning service; Maintenance/ Building management   - The Outsourcing Category 3/4 (*OC*3/4) is that the relationship between the C and SP belongs to a group in-between SP’s reliance/commitment and alliance/partner. - To justify this new group, it is essential to analyse the SC1 and SC2 of Ownership Substitution of FM Outsourcing strategies. The impacts of SC1 and SC2 as as high as that of Outsourcing Category 3 (*OC*3) and Category 4 (*OC*4). As for the Strategic Influence of FM Outsourcing strategies, the impacts of CP1 as in-between high and medium as Outsourcing category 1/2 (*OC*1/2) or Outsourcing category 2/3 (*OC*2/3). The impacts of LP1 as as high as that of Outsourcing Category 3 (*OC*3) and Outsourcing Category 4 (*OC*4) and the impacts of LP2 as high - When will the company fall on the Outsourcing Category 3/4 (*OC*3/4)? If the company is dynamically and marginally categorized at *OC*3, it is still having a progression to *OC*4. If this is the case, we would consider this company at the Outsourcing category 3/4 (*OC*3/4) (Commitment/ partner) which has a high impact on (SC1 and SC2) managerial control and decision making over operations, planning, development and implementation of facilities and personnel replacement in-house FM personnel, (CP1) competitive advantage and (LP1) competitiveness, but also has a high impact on (SC1 and SC2) and (LP1 and LP2) long-term competitiveness, a close partnership, strategic inter-organizational relationship and new revenue. - The SPs have the *OC*3 characteristic as follows. SPs can maintain monthly performance and value of customers’ satisfaction to health, safety, maintenance and operation management. - The SPs also have the *OC*4 characteristic as follows. The SPs can maintain yearly performance and value of customers’ satisfaction on emergency planning, business continuity and sustainability. | 3.6953.4103.3703.434 | 3/4 |
| 7 | Private (Building surveying consultancy)/ International/50 – 100/2/ Assistant building surveyor/ Building surveying consultancy |  |  |  |  |
| 8 | Private (Property management)/ regional/100 – 200/2/ Associate Director/ Provision of property management services | Security, cleaning, renovation | Comprehensive hard and soft PFM services   - ditto | 1.058  1.107 | 1 |
| X | X | X | - The Outsourcing Category 2 (*OC*2) is that the relationship between the C and SP belongs to a group in SP’s alignment/technical expertise. - The impacts of SO2 of the Ownership Substitution and CP2 of the Strategic Influence of FM Outsourcing strategies as quite high. - The SPs can provide at least sufficient or even higher standards of tailor-made professional knowledge for the building assets. - This is also that to be mutually agreed upon between the C and the SP for the confirmation of daily operations to the Cs’ requirements. The SPs can maintain a high level of customer’s general routine productivity on a high level for monthly operations. E.g., security management, waste management, cleanliness of the workplace and indoor environmental quality | X  X | 2 |

Table 2 – Raw data of the eight case studies of the seven outsourcing categories

| **Case Study** | **Industry** | **Officer** | **SO1** | **SO2** | **SC1** | **SC2** | **CP1** | **CP2** | **LP1** | **LP2** | **Input *OC*1** | **Input *OC*2** | **Input *OC*3** | **Input *OC*4** | **Output (raw)** | **Confirmed *OC*** |
| --- | --- | --- | --- | --- | --- | --- | --- | --- | --- | --- | --- | --- | --- | --- | --- | --- |
| 1 | Private (Building maintenance) | 1 (X) | 78.18 | 44.83 | 101.72 | 8.61 | 61.94 | 132.74 | 78.60 | 20.00 | **3.430** | **3.630** | 3.020 | 2.820 | 1.573 | *OC*1*/*2 |
|  |  | 2 (Y) | 87.27 | 62.07 | 89.44 | 4.73 | 60.00 | 137.76 | 68.84 | 20.00 | **3.450** | **3.650** | 2.770 | 2.630 | 1.449 | *OC*1*/*2 |
| 2 | Private (Property development) | 1 (X) | 63.64 | 100.00 | 78.94 | 81.23 | 66.45 | 98.72 | 64.47 | 10.00 | 3.810 | 4.020 | **3.950** | 3.340 | 3.261 | *OC*3 |
|  |  | 2 (Y) | 63.64 | 100.00 | 91.32 | 81.23 | 73.55 | 117.23 | 100.41 | 10.00 | 4.110 | 4.460 | **4.450** | 3.690 | 3.487 | *OC*3 |
| 3 | Public (Leisure and culture of Government) | 1 (X) | 58.18 | 20.69 | 100.00 | 100.00 | 63.87 | 92.33 | 73.06 | 75.79 | 3.560 | 3.560 | 4.000 | **4.010** | 3.889 | *OC*4 |
|  |  | 2 (Y) | 45.45 | 27.59 | 100.00 | 100.00 | 70.97 | 89.90 | 73.06 | 75.79 | 3.580 | 3.660 | 4.120 | **4.050** | 3.875 | *OC*4 |
|  |  | 3 (Z) | 60.00 | 20.69 | 100.00 | 100.00 | 66.45 | 88.66 | 73.06 | 75.79 | 3.590 | 3.560 | 4.010 | **4.020** | 3.907 | *OC*4 |
| 4 | Public (Construction & Maintenance) | 1 (X) | 81.82 | 48.28 | 86.85 | 34.86 | 72.90 | 94.76 | 88.40 | 13.20 | 3.650 | **3.730** | **3.590** | 3.160 | 2.529 | *OC*2*/*3 |
|  |  | 2 (Y) | 87.27 | 51.72 | 76.42 | 42.49 | 73.55 | 85.97 | 80.55 | 13.20 | 3.680 | **3.690** | **3.580** | 3.160 | 2.478 | *OC*2*/*3 |
| 5 | Public (Education & exhibition) | 1 (X) | 83.64 | 96.55 | 27.63 | 7.07 | 75.48 | 72.33 | 52.22 | 10.00 | **3.130** | 3.040 | 2.360 | 2.140 | 1.054 | *OC*1 |
|  |  | 2 (Y) | 81.82 | 82.76 | 35.02 | 6.15 | 72.26 | 73.80 | 60.46 | 10.00 | **3.090** | 3.060 | 2.390 | 2.170 | 1.147 | *OC*1 |
| 6 | Public (Property management) | 1 (X) | 70.91 | 62.07 | 100.00 | 81.23 | 76.77 | 82.33 | 118.91 | 23.52 | 4.090 | 4.270 | **4.500** | **4.020** | 3.695 | *OC*3*/*4 |
|  |  | 2 (Y) | 63.64 | 75.86 | 123.95 | 82.78 | 87.10 | 80.63 | 112.28 | 19.04 | 4.190 | 4.430 | **4.710** | **4.070** | 3.410 | *OC*3*/*4 |
| 7 | Private (Building surveying consultancy) | 1 (X) | 36.36 | 24.14 | 100.00 | 100.00 | 56.13 | 109.67 | 59.28 | 26.39 | 3.080 | 3.250 | **3.650** | **3.330** | 3.370 | *OC*3*/*4 |
|  |  | 2 (Y) | 34.55 | 20.69 | 100.00 | 100.00 | 77.42 | 112.42 | 59.28 | 31.04 | 3.210 | 3.310 | **3.800** | **3.440** | 3.434 | *OC*3*/*4 |
| 8 | Private (Property management) | 1 (X) | 12.73 | 55.17 | 5.00 | 4.78 | 24.52 | 123.49 | 48.32 | 25.51 | **1.760** | 1.940 | 1.520 | 1.510 | 1.058 | *OC*1 |
|  |  | 2 (Y) | 7.27 | 37.93 | 1.00 | 3.68 | 20.65 | 146.97 | 84.84 | 10.00 | **1.390** | 1.530 | 1.210 | 1.150 | 1.107 | *OC*1 |
